# Characterization of Aligned Polymers Using the Spin Hall Effect of Light

**DOI:** 10.3390/polym17070958

**Published:** 2025-03-31

**Authors:** Changyou Wang, Tong Li, Sijie Zhang, Zuhong Xiong, Zhiyou Zhang

**Affiliations:** 1 College of Mechanical Engineering, Guizhou University of Engineering Science, Bijie 551700, China; 2 College of Physics, Sichuan University, Chengdu 610065, China; 2022222020057@stu.scu.edu.cn (C.W.); lt@stu.scu.edu.cn (T.L.); zhangzhiyou@scu.edu.cn (Z.Z.); 3 Chongqing Key Laboratory of Micro&Nano Structure Optoelectronics, School of Physical Science and Technology, Southwest University, Chongqing 400715, China

**Keywords:** aligned polymers, poly (3-hexylthiophene) films, the spin Hall effect of light

## Abstract

In this paper, we propose a scheme based on the spin Hall effect of light (SHEL) for characterizing aligned polymers. Poly (3-hexylthiophene) (P3HT) films were prepared via the solution shear coating method. The experimental results obtained by using SHEL indicated that the alignment of polymer chains could be enhanced by UV irradiation and was positively correlated with the irradiation time, which was consistent with the measurements of the UV–Vis spectrometer and the polarizing optical microscope (POM). Compared with other optical methods, such as POM that characterizes aligned samples using light intensity, the amplified shift in SHEL can significantly reduce technical noise and exhibit high sensitivity. Even for the weak alignment film, this method can still recognize its alignment and achieve a signal-to-noise ratio (SNR) of 30 dB. This renders SHEL a highly precise technique for studying aligned polymers, which is valuable for the development of organic electronics.

## 1. Introduction

Conjugated polymers, due to their advantages like low-temperature processability and solution-processibility, have attracted significant attention in diverse research fields, including organic field-effect transistors (OFETs), organic light-emitting diodes (OLEDs), organic photovoltaic cells, and chemical and biosensors. Compared with traditional inorganic semiconductors, polymer materials have lower costs, better thermoelectric performance, and better flexibility. However, polymer semiconductors show poor charge transport properties, resulting in inferior device performance, mainly because of issues such as low crystallinity (related to intra- and inter-chain interactions) and random alignment.

Organic electronic devices based on aligned films have better charge transport characteristics than ordinary organic semiconductor devices [1,2]. This is because in ordinary organic semiconductor devices, the organic semiconductor active layer, such as the organic semiconductor polymer film, is usually composed of many disordered small crystal regions; thus, the interaction between the polymer chains within the film is weak. This is not conducive to charge transport, resulting in low carrier mobility and low device performance. In the contrast, aligned polymers, such as one-dimensional nanowires, can effectively improve the charge transport characteristics. Therefore, the preparation and characterization of aligned polymers is of significance for the development of organic electronics.

There have been many approaches to characterize the aligned polymers, including Diffraction of X-Rays (XRD), Scanning Electron Microscope (SEM), Transmission Electron Microscope (TEM), Atomic Force Microscope (AFM), UV–Vis spectrophotometer, Polarizing Optical Microscope (POM), and ellipsometers. Among them, optical techniques (such as POM and ellipsometers) are usually realized by measuring the change in the light intensity induced by the optical rotation, which is proportional to the alignment of the polymer chains [3]. However, the light intensity is affected by experimental conditions such as the stability of the light source, the mechanical vibration of the platform, and the noise of the detector. Thus, the measurement precision is extremely limited.

The spin Hall effect of light (SHEL) can solve the problem of the precision limit that light intensity has met. This is because the physical parameters are measured by the shift of the beam centroid induced by SHEL. The centroid shift is the result of the integration of light intensity and position, so it is almost unaffected by fluctuations in light intensity. SHEL refers to the phenomenon that when a linearly polarized light beam is reflected or transmitted at an interface, the two spin components split perpendicular to the refractive index gradient. This effect was first observed experimentally using weak measurement [4]. After that, it has been applied to the study of metasurfaces [5,6,7], high energy physics [8,9], metal surface plasmas [10,11,12,13], semiconductors [14,15], free space [16], chiral materials [17,18,19], and magnetic films [20,21,22], since it greatly compresses the technical noises, and the amplified shift is very sensitive to the optical properties of the materials. SHEL has also been studied in polymers, and it has been improved that SHEL can be enhanced by tuning the birefringence in the polymers with uniaxial anisotropy [23,24]. Compared with the polymers fabricated by spin coating, aligned polymers exhibit biaxial anisotropy. In theory, SHEL also has the ability to study materials with biaxial anisotropy.

In this work, SHEL is used to characterize aligned polymers by measuring the optical rotation induced by the samples. We use the method of solution shear coating to prepare poly (3-hexylthiophene) (P3HT) films, and the solutions are irradiated by UV with the irradiation times of 0, 2 min, 4 min, 6 min, and 8 min, respectively. The measurement results of UV–Vis, POM and AFM proved that the alignment of the polymer chains is enhanced by UV irradiation, and relies on the irradiation time. We obtained the same experimental result by using SHEL. Compared with traditional optical methods like POM, this technique has higher sensitivity. It is valuable for the study of alignment polymers and the development of organic electronics.

## 2. Materials and Methods

### 2.1. Materials and Sample Preparation

#### 2.1.1. Materials

All chemicals were used without additional purification. P3HT (CAS number 104934-50-1) with an average molecular weight (Mw) of $10^{4}$–$10^{5}$ kDa was purchased from Xi’an Polymer Light Technology Corp., Xian, China. CHCl_3_ was purchased from Chengdu Kelong Chemical Co., Ltd., Chengdu, China.

#### 2.1.2. Preparation of Aligned P3HT Films

**Figure 1:**
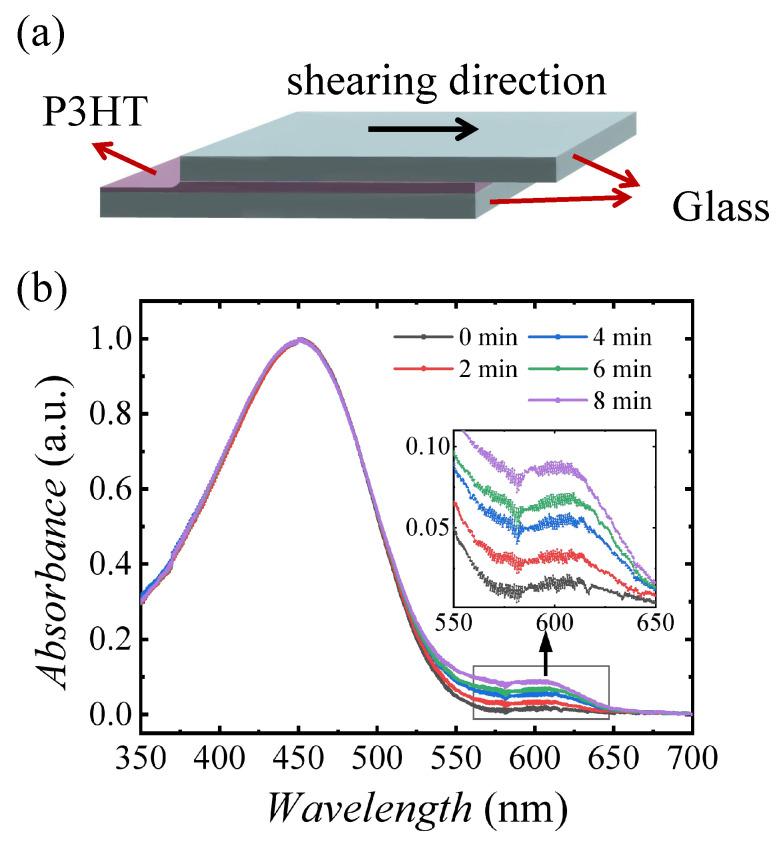
(**a**) Diagram of the process of solution shear coating; the black arrow marks the direction of shearing. (**b**) Measurement of the P3HT solutions with different UV–irradiation time, measured by UV–Vis spectrophotometer. The black, red, blue, green, and purple curves refer to the samples with the UV–irradiation times of 0, 2 min, 4 min, 6 min, and 8 min, respectively.

P3HT is a common polymer material capable of self-assembling to form a microcrystalline structure and has good solubility in various organic solvents. Therefore, P3HT is selected as the material for preparing the aligned polymer film. We prepare P3HT solutions with a concentration of 5 mg/mL using CHCl_3_ as the solvent. Then, we use a heating plate to accelerate the dissolution of P3HT in CHCl_3_ and set the temperature at 55 °C. Set the heating time for at least 20 min until P3HT is completely dissolved in CHCl_3_. After the solutions were cooled to room temperature, a Philips UV lamp (model MH-UV6W, power = 6W) was placed 15 cm above the solutions and directed downward to irradiate the solutions. The irradiation time was set to 0, 2, 4, 6, and 8 min. The solutions changed from bright orange to dark brown with increased UV irradiation time, indicative of an increase in the concentration of ordered aggregates [25]. Solution shear coating offers a simple yet effective approach to aligning conjugated polymer nanowires, thin films, and small molecules. The technique entails the controlled uniaxial shearing of a polymer semiconductor solution placed between two flat substrates, accompanied by slow solvent evaporation. Therefore, the solution shear coating method was chosen to prepare aligned P3HT. The solution shear coating process is shown in Figure 1a. We set the solution shear rate to 1 mm/s [1]. During the solution shearing process, the cover plate is dragged at a constant speed. Subsequently, the mixed solution is continuously sheared and mechanically deposited on the substrate, generating macroscopically aligned P3HT nanowires along the shearing direction.

### 2.2. Traditional Methods

#### 2.2.1. UV–Vis Spectroscopy

References show that UV irradiation can enhance the overlap of π orbitals and the planarization of the polymer chains, thus enhancing the orderly alignment of the molecules. And π−π packing between polymer chains affects anisotropic supramolecular assembly and enhances the formation of ordered aggregates [26]. Thereby, UV irradiation is used to improve the ordered alignment of P3HT polymers. The UV–Vis spectra of P3HT solutions with different UV–irradiation times were recorded using an Hanon i5 UV–Vis spectrometer purchased from Jinan Hanon Instruments Co., Ltd., Jinan, China.

#### 2.2.2. Atomic Force Microscopy (AFM)

The AFM measurements were performed on P3HT films using a Bruker Dimension Icon (Billerica, MA, USA) operating in tapping mode. The size of the scan area was set to 5 × 5 µm.

#### 2.2.3. Polarized Optical Microscopy (POM)

The POM images were obtained with PTF-150SZ purchased from Beijing Century Kexin Scientific Instruments Co., Ltd., Beijing, China. The magnification of the eyepiece and the objective lens is 10× and 5×, respectively. Initially, the two polarizers are 0 and 90° from the horizontal direction, respectively. At this time, the light intensity is 0 because the two polarizers are orthogonal to each other. Then, we put the sample on to observe the anisotropy of the sample using the brightness change induced by the polarization change in the light (optical rotation and circular dichroism). For an anisotropic sample, when the alignment direction (optical axis) is 0 or 90° from the horizontal direction, the changes in the brightness of the light reach the maximum. When the alignment direction is ±45° from the horizontal direction, the anisotropy does not induce the brightness change [1].

### 2.3. Method of SHEL

#### 2.3.1. Experimental Setup

**Figure 2:**
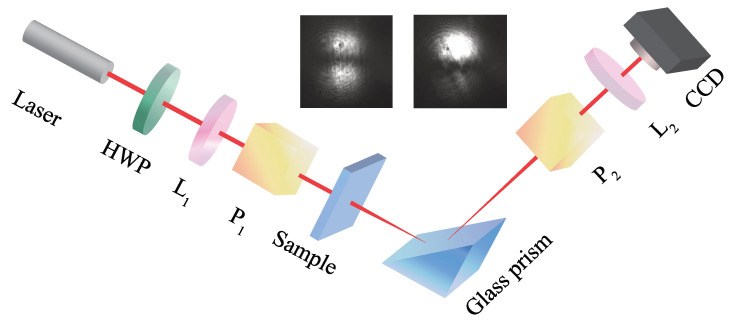
Measurement setup of SHEL. The light source is a He-Ne laser (wavelength = 632.8 nm), HWP is a half-wave plate, P_1_ and P_2_ are Glan laser polarizers, L_1_ and L_2_ are the lenses with focal lengths of 50 mm and 250 mm, respectively, CCD denotes the charge-coupled device. The inset shows the light spots received by CCD. The left and right inset photos in Figure 2 are recorded when the aligned sample is outside and inside the setup, respectively.

The measurement setup is shown in Figure 2. The light source is a He-Ne laser (wavelength = 632.8 nm, the part number is HNL210LB, purchased from Thorlabs, Newton, NJ, USA), which passes through a half-wave plate (HWP, wavelength = 632.8 nm, phase delay = λ/2, dimension diameter = 25.4 mm, part number is GCL-060713, purchased from Thorlabs) to adjust the intensity of the light. The lenses L_1_ (focal lengths = 50 mm, part number is GCL-010204, purchased from Beijing Daheng Photo-Electric Technology Company, Beijing, China.) and L_2_ (focal lengths = 250 mm, part number is GCL-010213, purchased from Beijing Daheng Photo-Electric Technology Company, Beijing, China.) are used to focus and collimate the beam both before and after the reflection off the surface. P_1_ and P_2_ are Glan laser polarizers (operating wavelength = 350–2500 nm, transmittance > 85% @ 632.8 nm, housing diameter = 25.4 mm, part number is GCL-070212, purchased from Beijing Daheng Photo-Electric Technology Company.), used to obtain the proper states, respectively. The detection of the reflected beam is carried out using a CCD photodetector (the charge-coupled device, part number is WP-UT400/M, purchased from Shenzhen Huagu Power Technology Co., Ltd., Shenzhen, China.).

Before the measurement, the CCD receives two opposite light spots with the same light intensity (the left inset photo in Figure 2). Then, the P3HT film is placed between P_1_ and the glass prism. The centroid of the spots shifted due to the optical rotation caused by the sample (the right inset photo in Figure 2). We tested the amplified shift at 0° and 45° for samples with different UV-irradiation time. Then the amplified shift is recorded 100 times for the evaluation of the measurement precision by CCD. Furthermore, we measured the change in the amplified shift within a small angular change to intuitively show the high sensitivity in characterizing the alignment films using SHEL. The sample we use is the P3HT film with a UV–irradiated time of 8 min. Initially, the alignment direction is 45° from the horizontal direction, where the amplified shift is 0. Then, we rotated the film while keeping the spot in the same position and recorded the amplified shift every 0.5°.

#### 2.3.2. Transmission in Aligned Polymers

Firstly, we discuss the transmission process in an aligned polymer. Since aligned polymers exhibit biaxial anisotropy, we can consider them as birefringent crystals. Considering a Gaussian beam with the polarization state of $|H\rangle =\frac{1}{\sqrt{2}}(|+\rangle +|-\rangle )$, prepared by the polarizer P_1_, whose transverse electric field can be written as

(1) $$\left[\begin{array}{c} E_{0}^{H}\\ E_{0}^{V} \end{array}\right]=\left[\begin{array}{c} 1\\ 0 \end{array}\right],$$

that is transmitted through a medium of a polymer sample with ordered alignment chains, the state of the transmitted light can be characterized by the following Jones equation

(2) $$\left[\begin{array}{c} E_{i}^{H}\\ E_{i}^{V} \end{array}\right]=M^{\prime }\left(\theta \right)\left[\begin{array}{c} E_{0}^{H}\\ E_{0}^{V} \end{array}\right]=\left[\begin{array}{c} \cos\gamma \\ \sin\gamma \end{array}\right],$$

where γ represents the optical rotation angle induced by the aligned polymer. Here, we ignored the circular dichroism since it contributes very little to the amplified shift of SHEL when γ is very small. Assuming that there is an angle θ between the optical direction of the medium and the X-axis of laboratory coordinates, the Jones matrix $M^{\prime }\left(\theta \right)$ that describes the polarization variation after passing through the polymer (with a thickness of *d*) can be expressed as

(3) $$\begin{array}{cc} M^{\prime }\left(\theta \right) & =R(-\theta )MR(\theta )\\ \; & =\left(\begin{array}{cc} \cos\theta & -\sin\theta \\ \sin\theta & \cos\theta \end{array}\right)(\begin{array}{cc} e^{-i\frac{\Delta \varphi }{2}} & 0\\ 0 & e^{i\frac{\Delta \varphi }{2}} \end{array})\left(\begin{array}{cc} \cos\theta & \sin\theta \\ -\sin\theta & \cos\theta \end{array}\right)\\ \; & =\left(\begin{array}{cc} \cos^{2}\theta \;e^{-i\frac{\Delta \varphi }{2}}+\sin^{2}\theta \;e^{i\frac{\Delta \varphi }{2}} & 2\sin\theta \cos\theta \sinh(-i\frac{\Delta \varphi }{2})\\ 2\sin\theta \cos\theta \sinh(-i\frac{\Delta \varphi }{2}) & \sin^{2}\theta \;e^{-i\frac{\Delta \varphi }{2}}+\cos^{2}\theta \;e^{i\frac{\Delta \varphi }{2}} \end{array}\right). \end{array}$$

where Δφ is the phase difference caused by the propagation of the ordinary light and the extraordinary light [27], which is defined as $\Delta \varphi =\frac{2\pi }{\lambda }\left(n_{e}-n_{o}\right)d$, where $n_{e}$ ($n_{o}$) are the refraction index of the polymer for the extraordinary (ordinary) light waves, respectively. From the expression of $M^{\prime }$, we can see that the state of the Gaussian beam after passing through the sample is affected by Δφ and θ. Since we only consider the case where the alignment direction of the polymer sample is parallel to the X-axis of laboratory coordinates (i.e., θ=0). The Jones matrix $M^{\prime }\left(\theta \right)$ can be simplified to

(4) $$M^{\prime }\left(\theta \right)=M=\left[\begin{array}{cc} e^{-i\frac{\Delta \varphi }{2}} & 0\\ 0 & e^{i\frac{\Delta \varphi }{2}} \end{array}\right],$$

Thus, substituting Equation (4) into Equation (2), we can see that $\gamma =\frac{1}{2}\Delta \varphi$ [28], the optical parameters and the information of the aligned polymer establish the following relationship:

(5) $$\gamma =\frac{\pi }{\lambda }\left(n_{e}-n_{o}\right)d.$$

So, we can see that γ is linearly related to $\left(n_{e}-n_{o}\right)$, the refractive index difference between the ordinary light and the extraordinary light. That is, the stronger the anisotropy caused by the orderly alignment of polymers, the larger the optical rotation angle.

#### 2.3.3. Process of SHEL

After passing through the alignment polymer sample, the polarization state of the beam can be expressed as

(6) $$\phi_{i}=\cos\gamma |H\rangle +\sin\gamma |V\rangle .$$

Then, the light is reflected from a glass prism with an incident angle of 45°, where SHEL occurs. This process can be expressed as

(7) $$\left[\begin{array}{c} E_{r}^{H}\\ E_{r}^{V} \end{array}\right]=\left[\begin{array}{cc} r_{p} & \frac{k_{y}\left(r_{p}+r_{s}\right)\cot\theta_{i}}{k_{0}}\\ -\frac{k_{y}\left(r_{p}+r_{s}\right)\cot\theta_{i}}{k_{0}} & r_{s} \end{array}\right]\left[\begin{array}{c} E_{i}^{H}\\ E_{i}^{V} \end{array}\right],$$

$r_{p}$ and $r_{s}$ are the Fresnel refractive indices of the glass prism.

Thus, we can obtain the polarization state of the whole system of the reflected light

(8) $$\begin{array}{cc} |\Phi_{r}\rangle = & \{\left[r_{p}\cos\gamma +\cot\theta_{i}\frac{k_{y}}{k_{0}}\left(r_{p}+r_{s}\right)\sin\gamma \right]|H\rangle \\ \; & +\left[-\cot\theta_{i}\frac{k_{y}}{k_{0}}\left(r_{p}+r_{s}\right)\cos\gamma +r_{s}\sin\gamma \right]\left|V\rangle \}\right|\phi_{p}\rangle \\ \approx & \exp\left(-i\delta {\hat{\sigma }}_{3}k_{y}\right)|\phi_{i}\rangle |\phi_{p}\rangle , \end{array}$$

where, ${\hat{\sigma }}_{3}=|+\rangle \langle +|-|-\rangle \langle -|$ is the spin operator of a photon. $\delta =\left(1+r_{s}/r_{p}\right)\cot\theta_{i}/k$ represents the spin splitting. $|\phi \rangle =\int dk_{y}|k_{y}\rangle {\left(w/\sqrt{2\pi }\right)}^{1/2}\exp\left(-w^{2}k_{y}^{2}/4\right)$ is the transverse distribution of the Gaussian beam, where *w* is the beam waist. And

(9) $$|\phi_{r}\rangle =\frac{1}{\sqrt{2}}(e^{-i\frac{r_{s}}{r_{p}}\gamma }|+\rangle +e^{i\frac{r_{s}}{r_{p}}\gamma }\left|-\rangle \right)$$

is regarded as the pre-selected state of the system. Where, γ represents the change in the polarization state induced by the sample. $|+\rangle =\frac{1}{\sqrt{2}}(|H\rangle +i|V\rangle )$ and $|-\rangle =\frac{1}{\sqrt{2}}(|H\rangle -i|V\rangle )$ are the left and right circularly polarized light, respectively.

We use a polarizer P_2_ to prepare the post-selected state, where

(10) $$\phi_{f}=\frac{1}{\sqrt{2}}(|+\rangle -|-\rangle ).$$

Thus, the polarization of the whole state can be calculated by

(11) $$|\Phi \rangle =\int \exp\left(-ik_{y}^{2}z/2k\right)dk_{x}dk_{y}|\phi_{p}\rangle |k_{x}\rangle |k_{y}\rangle |\phi_{f}\rangle \langle \phi_{f}|\phi_{r}\rangle ,$$

where $\exp(-ik_{y}^{2}z/2k)$ represents the free evolution of the beam (*z* is the free propagation distance). Finally, the amplified shift recorded by CCD is given by

(12) $$\langle y\rangle =\frac{\int \Phi^{*}\left(k_{y}\right)i\partial k_{y}\Phi \left(k_{y}\right)dk_{y}}{\int \Phi^{*}\left(k_{y}\right)\Phi \left(k_{y}\right)dk_{y}}.$$

when it satisfied the condition of $\Delta k_{y}\left|\delta \right|/\left|\gamma \right|\gg 1$. Equation (12) can be simplified to

(13) $$\langle y\rangle \approx \frac{2zr_{s}}{k\delta r_{p}}\gamma .$$

**Figure 3:**
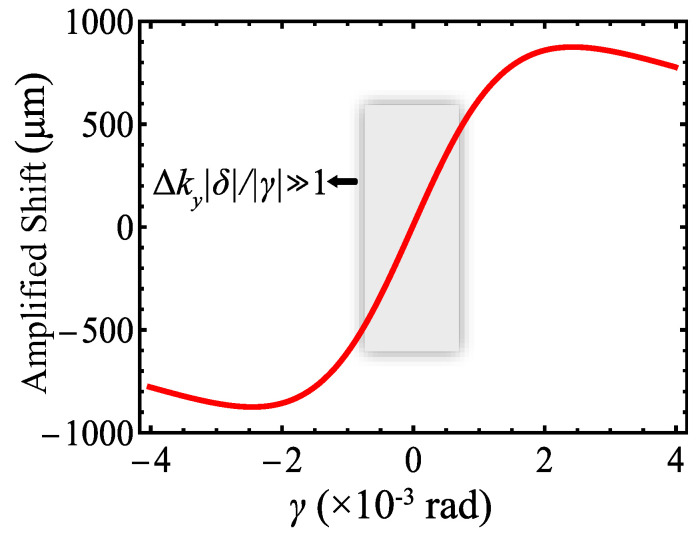
The theoretical amplified shift 〈y〉 as a function of γ. The gray region shows the linear response region for measurement.

The theoretical amplified shift 〈y〉 as a function of γ is shown in Figure 3. It can be seen that 〈y〉 is linearly related to the optical rotation induced by the sample when γ is small enough (the grey region). Since 〈y〉 is linearly related to the optical rotation induced by the sample, we can see that $\langle y\rangle \propto \left(n_{e}-n_{o}\right)$. Thus, 〈y〉 can be used to directly characterize the alignment polymers.

## 3. Results and Discussion

### 3.1. Characterization of UV-Vis Spectroscopy

The solutions with different irradiated times are characterized by UV–Vis spectrophotometer, as shown in Figure 1b. It shows all the solutions have a peak at 450 nm, corresponding to the typical high-energy π−π band transition peak. Additionally, two low-energy vibronic peaks near 570 nm and 620 nm are observed, attributed to the vibronic bands associated with the (0-1) and (0-0) transitions, respectively. The intensity of these vibronic peaks increases with UV–irradiation time, indicating enhanced molecular ordering and aggregation in the solution. UV irradiation promotes the planarization of P3HT chains and enhances the formation of ordered aggregates via π−π stacking between polymer chains in solution [26].

### 3.2. Characterization of POM

**Figure 4:**
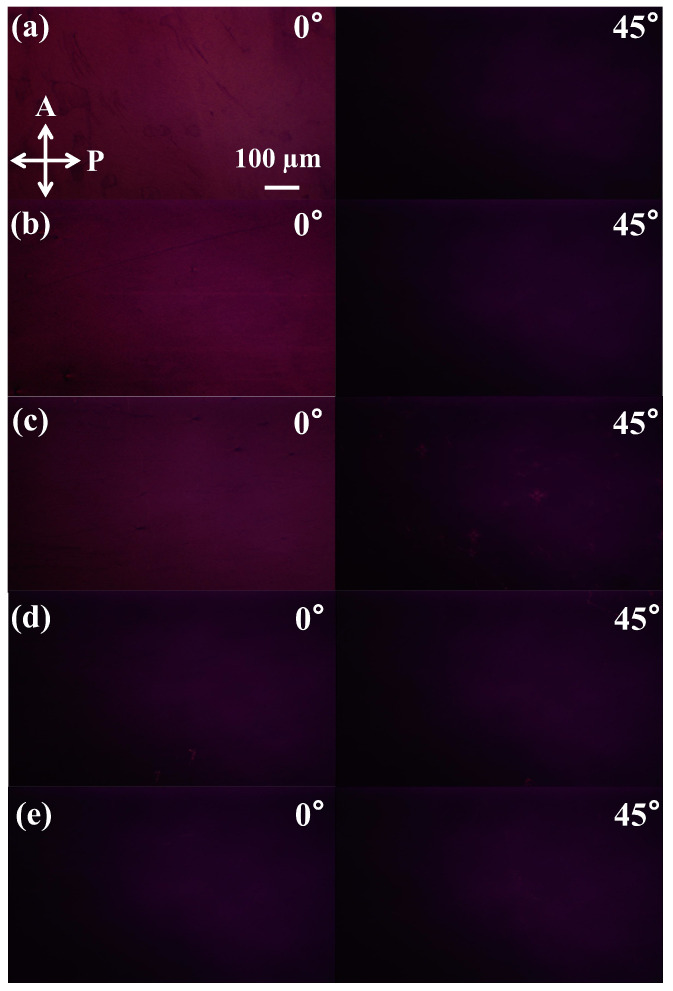
POM images of the P3HT films with different UV–irradiation time. The UV–irradiation times are (**a**) 8 min, (**b**) 6 min, (**c**) 4 min, (**d**) 2 min, and (**e**) 0. All images were taken with crossed polarizers. P and A indicate the axes of the microscope polarizer and of the light vibration plane, respectively.

The photos of the samples with the shearing direction along and at 45° from the horizontal direction are shown in Figure 4. It can be seen that all the photos are completely dark when the shearing direction is 45° from the horizontal direction. For the sample without UV irradiation and with the UV–irradiated time of 2 min, there is almost no change in the light intensity with the rotation of the shearing direction. For the samples with UV–irradiated time of 4, 6, and 8 min, when the shearing direction is along the horizontal direction, the light is getting brighter. And with the increasing of the irradiation time, the light intensity increases. Thus, we can infer that the anisotropy of P3HT films is enhanced by UV radiation, and the sample without UV–irradiation stays isotropic. It is because UV irradiation enhanced the formation of P3HT nanowires, and this process is positively correlated with the irradiated time, which caused the optical rotation and circular dichroism.

### 3.3. Characterization of AFM

**Figure 5:**
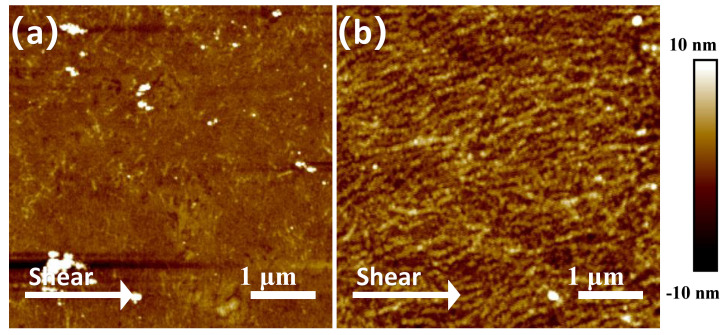
Tapping mode AFM phase images of (**a**) P3HT film without UV irradiation, and (**b**) P3HT film with UV irradiation for 8 min. The scan area size was set to 5 × 5 µm, and the white arrow marks the direction of shearing.

Atomic force microscopy (AFM) in tapping mode was performed on P3HT films. From Figure 5, we can see that compared with the P3HT films irradiated for 8 min, the films irradiated for 2–6 min are not well-aligned. So, we chose the 0 min (non-irradiated) and 8 min UV–irradiated samples for AFM analysis. The size of the scan area was set to 5 × 5 µm. The tested samples included P3HT films prepared from solutions without UV irradiation and those irradiated for 8 min. As shown in Figure 5a, the P3HT film derived from the non-irradiated solution shows an initial featureless and amorphous-like structure without aggregate formation. In contrast, Figure 5b demonstrates that the P3HT film prepared from the 8-min UV–irradiated solution exhibited well-aligned nanowire aggregates oriented predominantly along the solution shear coating direction. These results confirm that UV irradiation induces the formation of nanowire aggregates. The solution-based nanoaggregates appear to survive a solution shear coating process, successfully yielding aligned P3HT films.

### 3.4. Characterization of SHEL

**Figure 6:**
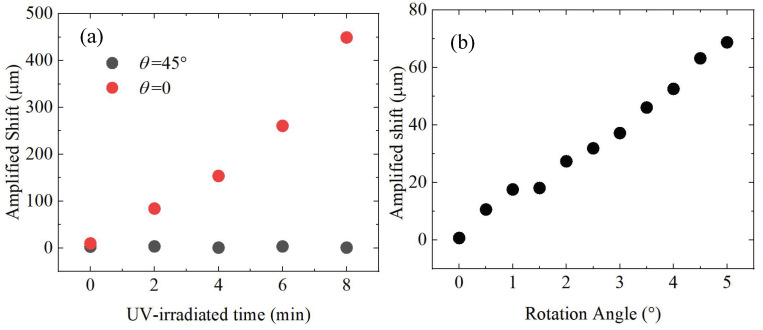
(**a**) The amplified shift as a function of solution UV–irradiation time, (**b**) The amplified shift as a function of the rotation angle of the sample with UV–irradiated time of 8 min.

The measurement result of SHEL is shown in Figure 6a. The red points are measured when the shearing direction is parallel to the horizontal direction, where the amplified shift reaches the maximum. The black points are measured when the shearing direction is 45° from the horizontal direction. It can be seen that there is a shift for the UV–irradiated P3TH films, while for the sample without UV irradiation, the amplified shift remains 0. With the increase of the UV–irradiation time, the amplified shift increases and achieves to 450 µm. This indicates the alignment direction is parallel to the shearing direction, and the alignment of the polymer chains is enhanced by the UV irradiation, which is consistent with the measurement results of POM. In contrast with POM, SHEL technique has higher sensitivity. Even for the weak alignment film (UV–irradiation time of 2 min), the SHEL can still recognize its alignment and achieve a signal-to-noise ratio (SNR) of 30 dB.

Furthermore, the amplified shift as a function of the rotation angle of the sample with UV–irradiated time of 8 min is shown in Figure 6b. We can see an obvious shift even in small angle of rotation, and the amplified shift reaches 75 µm when the rotation angle is 5°. The measurement precision of the amplified shift is calculated based on 100 measurements. In this experiment, a standard deviation of 0.8 µm is achieved (far less than the signal). Thus, we can estimate that we can measure the variation of the amplified shift caused by a rotation angle of 0.05° using error propagation theory.

## 4. Conclusions

In conclusion, we propose to use the amplified shift of SHEL to characterize the aligned polymers by establishing the relationship between the alignment-induced optical rotation and the amplified shift. Using this method, we experimentally observed an enhancement of the alignment of the polymer chains. And the precision of 0.8 µm is achieved for measuring the amplified shift. We came to the same conclusion using the UV–Vis spectrophotometer, POM, and AFM. Compared with the traditional optical methods like POM that use the change in light intensity to observe the alignment of polymers, this method relies on centroid shifts of light beams for measuring physical parameters, significantly compresses the technical noises, and has higher sensitivity, which is valuable for further study of organic polymer materials and devices.

## Data Availability

The original contributions presented in this study are included in the article. Further inquiries can be directed to the corresponding authors.
